# The effect of intermittent hypoxia and fecal microbiota of OSAS on genes associated with colorectal cancer

**DOI:** 10.1007/s11325-020-02204-z

**Published:** 2020-10-07

**Authors:** Jia Gao, Hailong Cao, Qiang Zhang, Bangmao Wang

**Affiliations:** 1 Department of Gastroenterology and Hepatology, Tianjin Medical University General Hospital, No.154, Anshan Road, Heping District, Tianjin, China; 2 Department of Geriatrics, Tianjin Geriatrics Institute, Tianjin Medical University General Hospital, No.154, Anshan Road, Heping District, Tianjin, China

**Keywords:** Colorectal cancer, Obstructive sleep apnea syndrome, Intermittent hypoxia, Intestinal microbiota

## Abstract

**Purpose:**

Colorectal cancer (CRC) is one of the common causes of cancer death worldwide. Obstructive sleep apnea syndrome (OSAS), sharing many risk factors in common with CRC, is prevalent among CRC patients. OSAS may promote the CRC development independently but the mechanism is still unknown. Intermittent hypoxia (IH) is one of the characteristics of OSAS, and hypoxia may influence the genes associated with CRC. Intestinal microbiota plays important role in CRC carcinogenesis, and OSAS patients have been shown to have intestinal microbiota dysbiosis. We hypothesized that IH and intestinal microbiota dysbiosis may be involved for CRC in patients with OSAS.

**Methods:**

We established precancerous cell models of CRC with Immorto-Min colonic epithelial (IMCE) cells. First, the cells were exposed to IH in a special chamber for 4 h, 8 h, and 12 h. Feces from 6 patients with OSAS and 6 healthy controls were collected and made into sterile fecal fluid for incubation with IMCE cells for 12 h. The cells were then exposed to IH for 4 h, 8 h, and 12 h. After IH exposure, the expressions of genes and inflammation cytokines associated with CRC, such as β-catenin, STAT3, HIF-1α, IL-6, TNF-α, c-myc, and cyclinD1, were tested.

**Results:**

IH activated the expression of HIF-1α and STAT3 both in mRNA and protein level (HIF-1α: *P* = 0.015 for mRNA level, *P* = 0.027 for protein level; STAT3: *P* = 0.023 for mRNA level, *P* = 0.023 for protein level), and promoted p-STAT3 shifting to the nucleus (*P* = 0.023). The mRNA of β-catenin (*P* = 0.022) and cyclinD1 (*P* = 0.023) was elevated, but there was no change for the β-catenin protein in the nucleus. Gut microbiota of OSAS patients promoted the expression of STAT3 (protein level: 0 h: *P* = 0.037; 4 h: *P* = 0.046; 8 h: *P* = 0.049; 12 h: *P* = 0.037), promoted p-STAT3 (4 h: *P =* 0.049; 8 h: *P =* 0.046; 12 h: *P =* 0.046) shifting to the nucleus, and also elevated the expression of IL-6 and TNF-α in mRNA level at 4 h (IL-6: *P* = 0.037, TNF-α: *P* = 0.037) and 8 h (IL-6: *P* = 0.037, TNF-α: *P* = 0.037). The protein of β-catenin in the nucleus was not affected by IH and gut microbiota from OSAS.

**Conclusions:**

Our study demonstrated that IH and gut microbiota of patients with OSAS activated HIF-1α expression and STAT3 pathway in IMCE cells, with no influence on β-catenin pathway, which suggested that IH, STAT3 pathway, chronic inflammation, and intestinal microbiota dysbiosis may be involved in CRC carcinogenesis correlated with OSAS These findings must be interpreted cautiously and further research is necessary to clarify the causative steps in CRC development.

## Introduction

Colorectal cancer (CRC) is very common worldwide, which lists third in morbidity and second in mortality overall worldwide [1], and the trend is still uprising in many countries, such as Russia, China, and Brazil. Twenty percent emergence of CRC could be referred to genetic background, such as hereditary non-polyposis colorectal cancer (HNPCC or Lynch syndrome), hamartomatous polyposis syndrome, and familial adenomatous polyposis (FAP), with characteristic of family history. The largest fraction of CRC cases is linked to environmental and nonhereditary events. Among them, chronic inflammation is a significant risk factor for CRC development [2], and some cytokines such as IL-6 and TNF-α play very important role in this process. The activations of Wnt/β-catenin pathway and IL-6/STAT3 pathway have been proved key procedures in CRC carcinogenesis. The environment pathogenic factors of CRC are commonly about personal lifestyle, and the relevant research is useful for disease prevention. High fat diet, insufficient intake of dietary fiber, high consumption of red or processed meat, body fatness, alcohol drinks, gut microbiota disorder [3], and ulcerative colitis [4] have been proved to have the relationship with CRC. People with these characteristics usually have obesity problem and many metabolism abnormalities, and also together with sleep apnea problems. Sleep apnea is indeed prevalent among colorectal cancer patients [5].

Obstructive sleep apnea syndrome (OSAS), also very prevalent in modern society, is a kind of sleep breathing disorder, with repeated partial or complete upper airway collapse. OSAS is characterized by intermittent hypoxia (IH), microarousal, and sleep fragmentation during sleep [6], affecting at least 2–4% of the adult population [7]. Many OSAS patients share the common risk factors with CRC patients: obesity body shape, often caused by unhealthy lifestyle. OSAS could induce multi-system disorders, and has been considered an independent risk factor for cardiovascular disease, cerebrovascular disease, and metabolic disease [8–16]. The patients with OSAS usually have elevated cancer burden [17–19]. In patients with lung cancer for example, OSAS is very prevalent [20, 21]. Some studies also have shown that OSAS could increase the risk of developing colorectal cancer [22, 23]. A cohort study suggested that OSAS even may promote the CRC development independently beyond the obesity [24]. These studies have demonstrated the association between OSAS and CRC, but the mechanism has not been investigated.

Intermittent hypoxia (IH) is key characteristic of OSAS, and it could induce multiple organ impairment. Continuous hypoxia condition could promote colon cancer cell proliferation and invasion [25–27], but whether IH could induce the carcinogenesis of CRC is still not clear and lack of related research. Beside the direct hypoxia effect, IH could activate the hypoxia-induced factor (HIF) and NF-κB, promoting the release of inflammatory factor [28], such as IL-6 and TNF-α. Elevated levels of IL-6 [29] and TNF-α [30] have been observed in OSAS patients, which may exert effect on intestinal epithelial cells and induce the inflammation and carcinogenesis in the intestine. Hypoxia and HIFs could also influence both Wnt/β-catenin and IL-6/STAT3 pathways. But whether these hypotheses work in CRC carcinogenesis needs further investigation.

The human large intestine contains the most microorganisms in the body, which play an important role in absorption, metabolism, and storage of ingested nutrients, with potentially profound effects on host physiology. Intestinal microbiota could also influence the inflammation level through interaction with immunocyte and producing short-chain fatty acid (SCFA) by fermentation. Microbiota dysbiosis has been proved to be associated with CRC [31]. Some specific bacteria, such as Bacteroides fragilis and Fusobacterium nucleatum, could impose effect on E-cadherin and activate Wnt/β-catenin signaling by toxin or adhesin, promoting colorectal carcinogenesis [32, 33]. These two kinds of pathogenic bacteria are both anaerobic bacteria, which may show more adaptation for the hypoxia microenvironment in OSAS patients compared with common intestinal bacteria. Some research has shown that OSAS could induce the intestinal microbiota dysbiosis [34], and microbiota dysbiosis correlation with OSAS could induce the inflammation [35, 36]. Then, we could assume that the intestinal microbiota dysbiosis in OSAS patients may contain more pathogens or induce the inflammation in the intestine, which could induce the CRC carcinogenesis.

Therefore, we hypothesized that IH and intestinal microbiota dysbiosis in OSAS patients may have the ability to activate one or more CRC carcinogenesis pathways, and inflammation may be involved in this process. To validate our hypotheses, we exposed Immorto-Min colonic epithelial (IMCE) cells to IH and sterile fecal supernatant from OSAS to establish precancerous cell model, mimicking CRC premalignancy in OSAS patients, and the expressions of genes and inflammation cytokines associated with colorectal cancer, such as β-catenin, STAT3, HIF-1α, IL-6, TNF-α, c-myc, and cyclinD1, were analyzed to evaluate the effect of IH and microbiota of OSAS on CRC development.

## Materials and methods

### IMCE cell culture and IH exposure

IMCE cell line was preserved in the laboratory of Gastroenterology and Hepatology department (Tianjin Medical University General Hospital), which was kindly provided by Professor Fang Yan from Vanderbilt University. IMCE cells were cultured in RMPI 1640 medium (Gibco, Invitrogen Corporation, NY, USA) supplemented with 10% FBS (Gibco, Invitrogen Corporation, NY, USA), 0.05% interferon-γ, 100 U/ml benzyl penicillin, and 100 μg/ml streptomycin under a circumstance of 33 °C with 5% CO2, and passaged every 3–5 days. Before IH exposure, the cells were changed into starvation medium (RMPI 1640 medium mixed with 1% FBS, 100 U/ml benzyl penicillin, and 100 μg/ml streptomycin) for 12 h. Then, cells were exposed in the experiment room, under intermittent hypoxic condition for 4, 8, or 12 h, and parts of cells were preserved without IH treatment as the control group. IH exposure was in a plexiglas chamber, which was alternately flushed with a hypoxia gas mixture (1.5% O2, 5% CO2, and balanced N2, hypoxia phase, 300 s) or normoxia gas mixture (21% O2, 5% CO2, and balanced N2, reoxygenation phase, 600 s) controlled by a computed engine. This IH exposure system for cell experiments was set up earlier by the Department of Respiratory Medicine in Tianjin Medical University General Hospital [37], and it has been widely used for OSAS simulation [38–40]. Our research team has used this system to mimic OSAS impairment for hippocampal neurons [41], pancreatic β-cells [42], and liver cells [43].

### Cell incubation with feces fluid of OSAS patients

Fresh feces were collected from 6 OSAS patients (O group) and 6 control healthy people (C group). In each group, 1 g of each sample was taken to mix together. The mixed feces were placed in sterile phosphate-buffered saline (100 mg/ml), homogenized, and centrifuged at 1000 rpm for 1 min, and the supernatant was collected as previously described [44, 45]. Then, the fecal fluid was filtrated through a 40-μm filter (BD Falcon) to remove big impurities. After that, a 0.2-μm filter (Acrodisc syringe filter) was used to filter the bacteria in the fecal supernatant. Twelve hours before incubation, the cells were changed into starvation medium. Then, filtered fecal supernatant was added into cell culture medium (1:100) to incubate, under a circumstance of 37 °C for 12 h. Then, the cells were treated under intermittent hypoxic condition for 4, 8, or 12 h. The ways of fecal fluid preparation and incubation with intestinal cells were used earlier for research of adenocarcinoma caused by dysbiosis [44], and for the research of chronic constipation caused by dysbiosis [46], but fecal fluid in former experiment was not suitable for longtime incubation with cells because it was not sterile; here we improved the way.

### Real-time PCR analysis

Total RNA was extracted by Trizol reagent (Applied Biosystems, USA), and cDNA reverse transcription was carried out using the TIANScript RT kit (TIANGEN, Inc. Beijing, China) according to the manufacturer’s instructions. The oligonucleotide primers were synthesized in GENEWIZ (Suzhou, China). Relevant oligonucleotide primer sequences were shown in Table 1. All the primer sequences were validated in nucleotide BLAST (http://blast.ncbi.nlm.nih.gov/Blast.cgi). Real-time PCR was conducted to quantify the transcription of cytokines such as IL-6 and TNF-α, and the transcription of genes associated with colorectal cancer such as HIF-1α, β-catenin, STAT3, cyclinD1, and c-myc. Glyceraldehyde-3-phosphate dehydrogenase (GAPDH), known as a housekeeping gene, was used as inner control to normalize the relative expression of targeted genes at mRNA level, which has been validated in some former researches [47–55]. ABI Prism 7000 real-time PCR system (Applied Biosystems, Carlsbad, CA) was used for real-time PCR procedure. SYBR Green PCR Master Mix (SYBR Select Master Mix, Applied biosystems, USA) was used for the RT-PCR detection. The procedure of PCR was constituted of 30 cycles followed by a period of 5 min at 72 °C for final extension. Within each cycle, the time period and temperature were 94 °C for 30 s, 60 °C for 30 s, and 72 °C for 90 s, respectively. The 2^−ΔΔCt^ method was used to calculate relative mRNA expression.

**Table 1:** Gene sequences of primers in the present study

Primers	Sequences
GAPDH	Forward: 5′-TGTGTCCGTCGTGGATCTGA-3′ Reverse: 5′-CCTGCTTCACCACCTTCTTGA-3′
HIF-1α	Forward: 5′-TGCCACTTCCCCACAATG-3′ Reverse: 5′-GTCCATCTGTGCCTTCATCTC-3′
IL-6	Forward: 5′-CCAGTTGCCTTCTTGGGACT-3′ Reverse: 5′-GGTCTGTTGGGAGTGGTATCC-3′
TNF-α	Forward: 5′-ACTCCAGGCGGTGCCTATG-3′ Reverse: 5′-GAGCGTGGTGGCCCCT-3′
β-catenin	Forward: 5′-GACACCTCCCAAGTCCTTTATG-3′ Reverse: 5′-AGCCCTAGTCATTGCATACTG-3′
STAT3	Forward: 5′-GGCACCTTGGATTGAGAGTC-3′ Reverse: 5′-AGGACATTGGACTCTTGCAG-3′
cyclinD1	Forward: 5′-TGACTGCCGAGAAGTTGTG-3′ Reverse: 5′-TTGGAGAGGAAGTGTTCGATG-3′
c-myc	Forward: 5′-GCTGTTTGAAGGCTGGATTTC-3′ Reverse: 5′-GATGAAATAGGGCTGTACGGAG-3′

### Western blot analysis

After the IH treatment was finished, the cells were washed with phosphate-buffered saline (PBS) for two times, and then RIPA buffer was used before lysed. Nucleus and cytoplasmic protein extraction kit (Beyotime Biotechnology, Shanghai, China) was used to extract the protein from the cells. Proteinase inhibitor cocktail (10 μl/ml) (Sigma, St. Louis, MO, USA) and phosphatase inhibitor cocktail (10 μl/ml) (Sigma, St. Louis, MO, USA) were added separately. After that, the lysate was homogenized and centrifuged (12,000*g*, 4 °C, 15 min), and the supernatants were retained. The protein was separated by SDS-polyacrylamide gel electrophoresis, then transferred onto a PVDF membrane. Rabbit monoclonal antibodies (Abcam, USA), anti-β-catenin, anti-HIF-1α, anti-IL-6, anti-p-STAT3, and anti-STAT3 were used correspondingly as primary antibodies. β-actin was used for total protein internal control and Histone 2A for nucleus protein internal control. Goat anti-rabbit IgG conjugated with horseradish peroxidase antibodies (Abcam, USA) was used as secondary antibody. A chemiluminescence detection system (BIO-RAD, USA) was used to detect the membranes. Comparison between the intensity of targeted bands and the intensity of internal control band was achieved via an image processor program (Image J 1.51j8).

### Statistical analysis

All statistical tests were performed with IBM SPSS Statistics 24. The data are described as mean ± SD. Statistical analyses were performed by using Mann-Whitney test in comparison between only two groups. Data were compared between multi-groups by Kruskal-Wallis *H* test. *P* value less than 0.05 was considered statistically significant difference.

## Results

### IH promoted HIF-1α expression

**Fig. 1:**
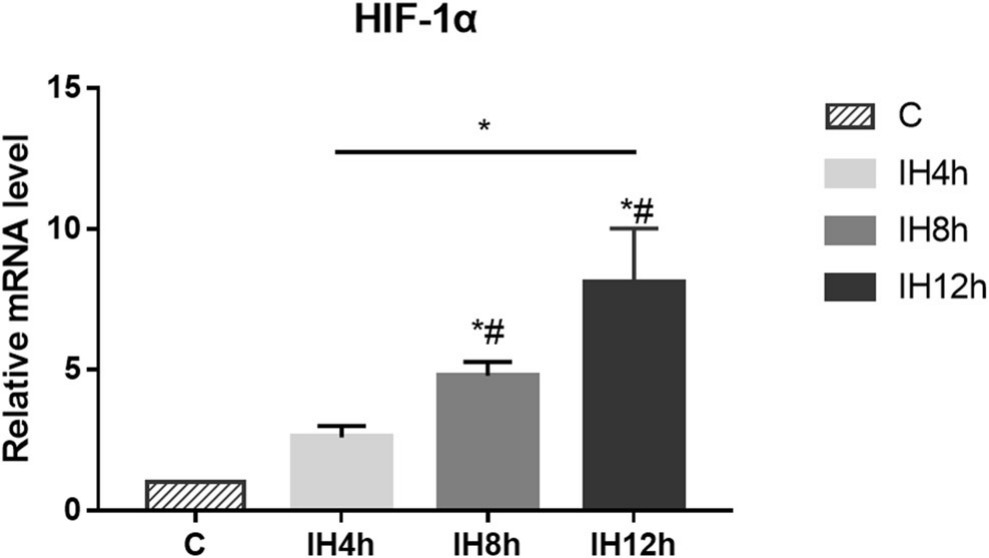
The effect of IH on HIF-1α expression in mRNA level. Kruskal-Wallis *H* test, Sig. = 0.015, *P < 0.05, *#*P* < 0.05 vs. C group, respectively

IH could promote HIF-1α expression both in mRNA level and protein level. We could observe that the mRNA level of HIF-α was significantly elevated after 8 h and 12 h IH treatment compared with the control group (C group) (*P* < 0.05, *F* = 27.819), and increased during the longer IH exposure, but there were no statistically significant differences in the levels between 4 and 8 h IH groups (Fig. 1). Similarly, the protein level of HIF-α was elevated after 12 h IH compared with the control group (*P* < 0.05, *F* = 31.818), though with increasing trend, but there were no statistically significant differences in the levels between 8 and 12 h IH groups (Fig. 4b). These results showed that IH could activate HIF-1α expression, and expression level correlated with length of IH exposure within 12 h.

### IH may induce IMCE cell proliferation

**Fig. 2:**
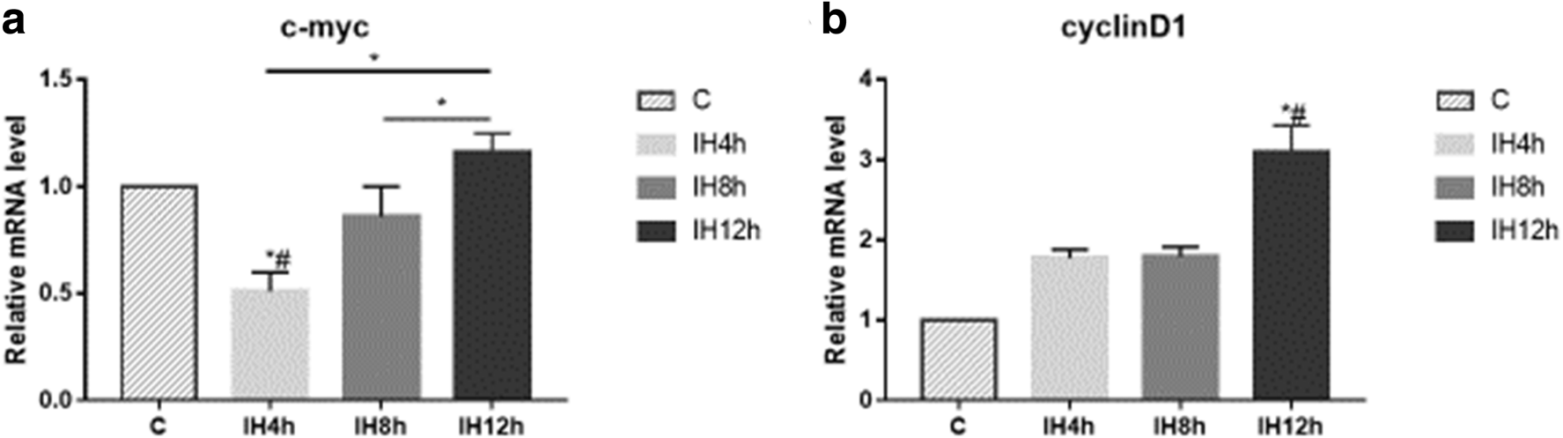
The effect of IH on expression of genes related to the control of cell proliferation: c-myc (**a**), cyclinD1 (**b**), detected by real-time-PCR. **a** Kruskal-Wallis *H* test, Sig. = 0.015. **b** Kruskal-Wallis *H* test, Sig. = 0.023. *P < 0.05, *#*P* < 0.05 vs. C group, respectively

The mRNA level of c-myc was downregulated after 4 h IH (*P* < 0.05, *F* = 25.7), then increased during the longer IH exposure, but there were no statistically significant differences on the levels of 8 h and 12 h IH groups compared with the control group (Fig. 2a). The expression of cylinD1 in mRNA level increased after 12 h IH compared with the control group (*P* < 0.05, *F* = 71.821); though with increasing trend, the levels of 4 h and 8 h group had no statistically significance compared with the control group (Fig. 2b). These results suggested that IH could activate cyclinD1 expression, which indicate cell proliferation. Though we did not see c-myc expression increased statistically compared with the control group, the results still showed the increasing trend during IH exposure.

### IH could activate tumorigenesis pathway

**Fig. 3:**
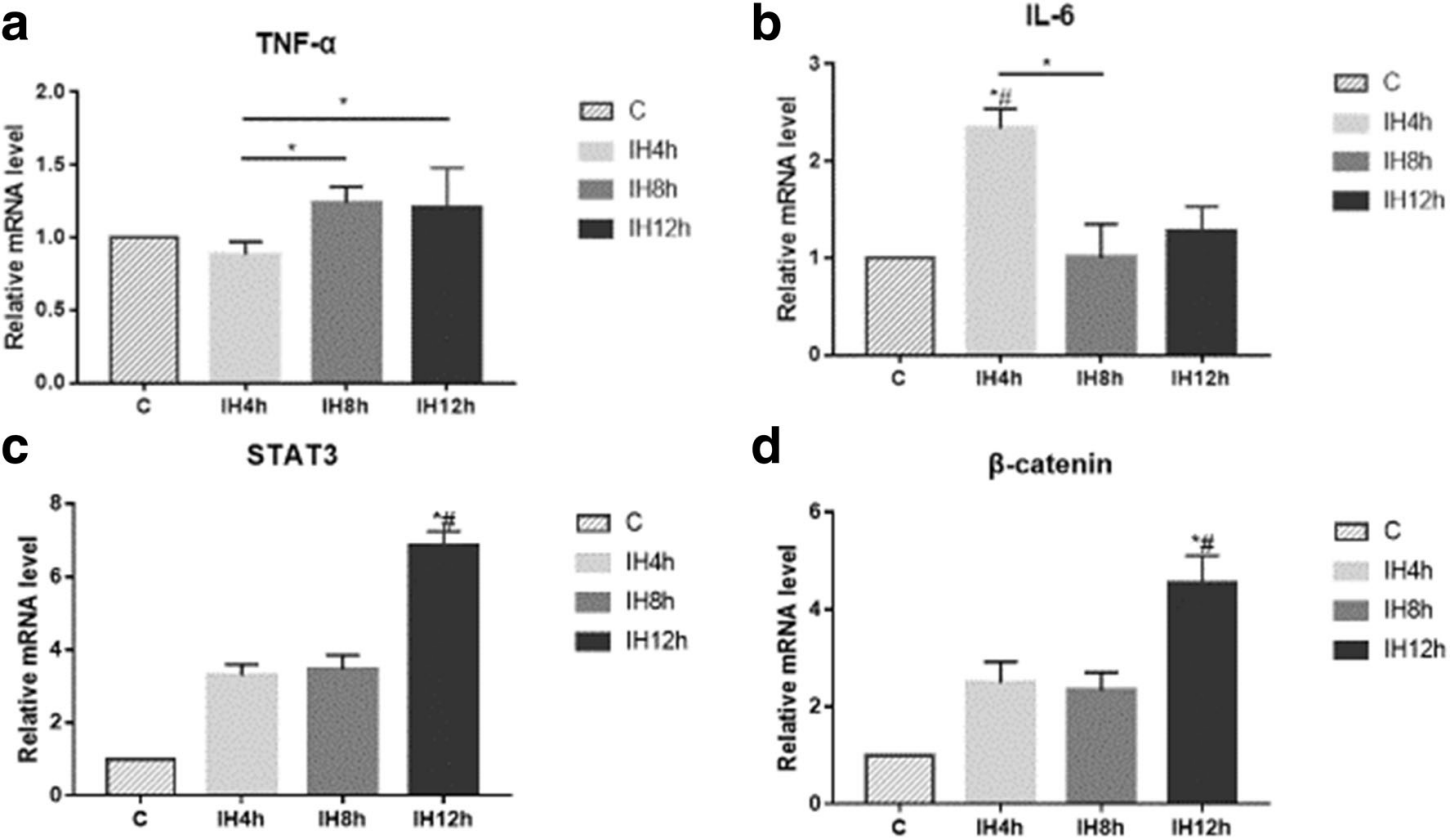
The effect of IH on CRC tumorigenesis pathway, mRNA level of TNF-α (**a**), IL-6 (**b**), STAT3 (**c**), and β-catenin (**d**), detected by real-time-PCR. **a** Kruskal-Wallis *H* test, Sig. = 0.047. **b** Kruskal-Wallis *H* test, Sig. = 0.041. **c** Kruskal-Wallis *H* test, Sig. = 0.023. **d** Kruskal-Wallis *H* test, Sig. = 0.022.*P < 0.05, *#*P* < 0.05 vs. C group, respectively

**Fig. 4:**
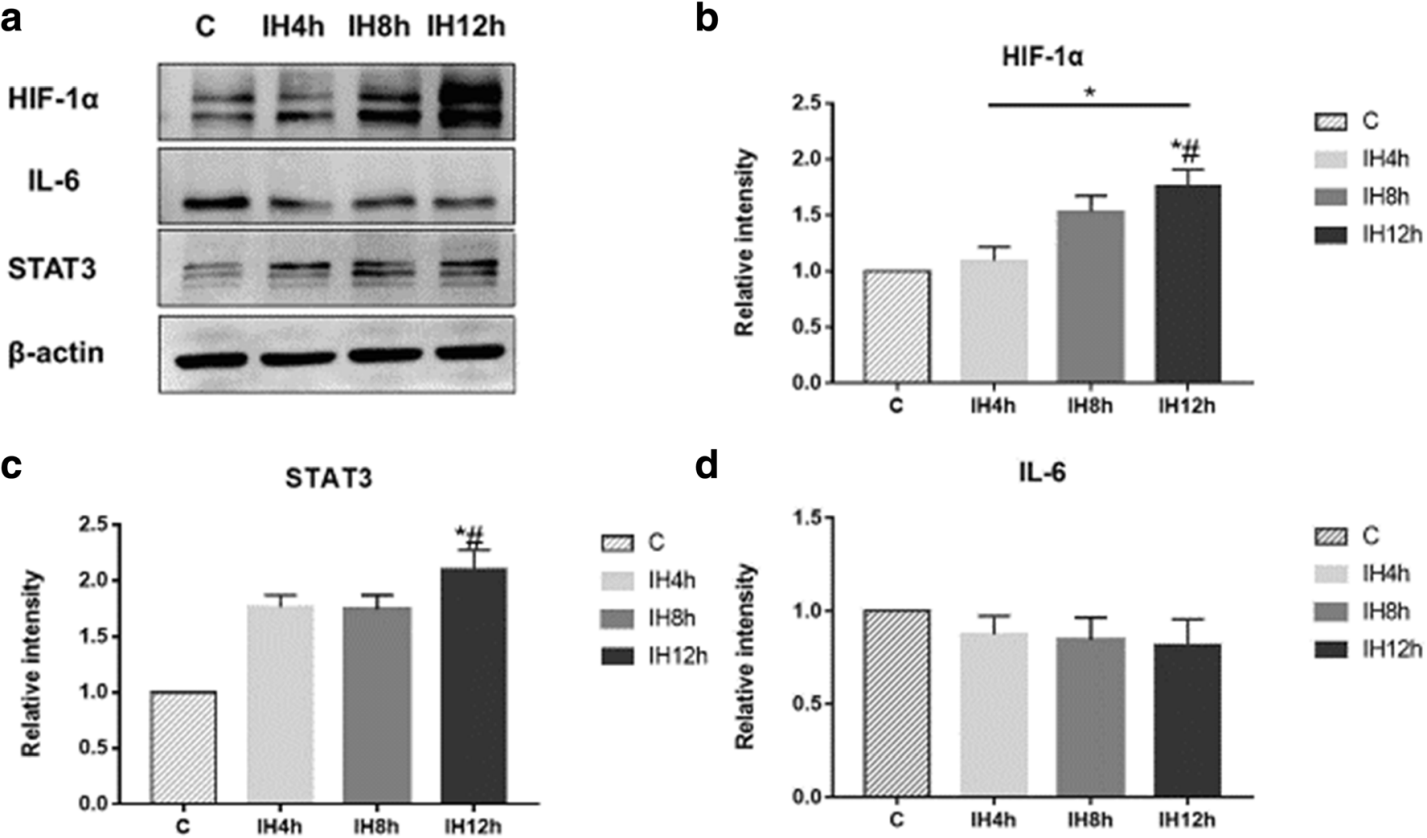
The effect of IH on CRC tumorigenesis pathway, detected by Western blot analysis (**a**): the protein level of HIF-1α (**b**), STAT3 (**c**), and IL-6 (**d**). **b** Kruskal-Wallis *H* test, Sig. = 0.027. **c** Kruskal-Wallis *H* test, Sig. = 0.023. **d** Kruskal-Wallis *H* test, Sig. = 0.55. *P < 0.05, *#*P* < 0.05 vs. C group, respectively

**Fig. 5:**
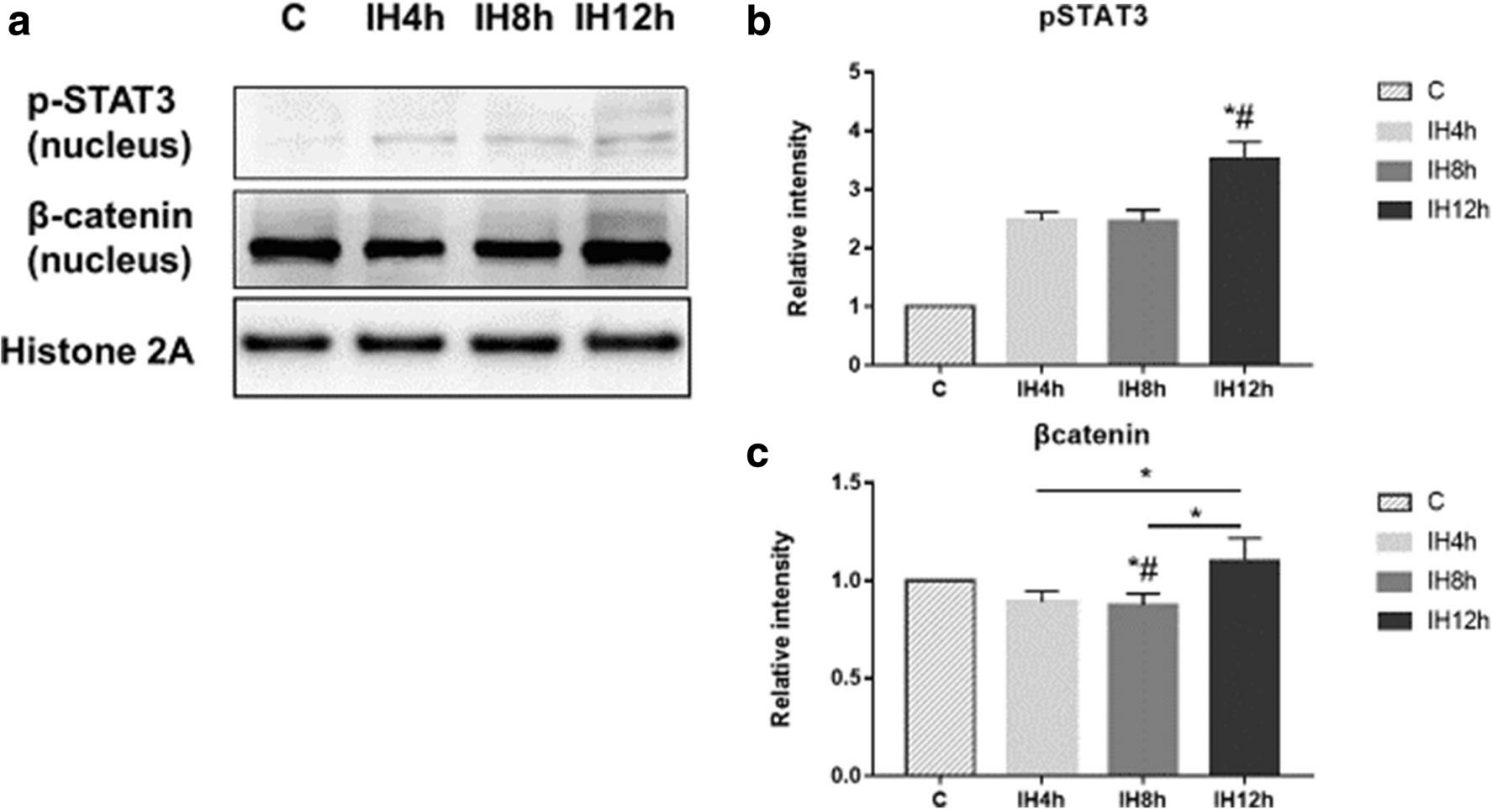
The effect of IH on CRC tumorigenesis pathway, detected by Western blot analysis (**a**): the protein level of p-STAT3 (**b**), β-catenin (**c**), in IMCE cell nucleus. **b** Kruskal-Wallis *H* test, Sig. = 0.023. **c** Kruskal-Wallis *H* test, Sig. = 0.034. *P < 0.05, *#*P* < 0.05 vs. C group, respectively

β-catenin was detected in mRNA level, and protein level in the nucleus to observe whether Wnt/β-catenin pathway was activated, together with detecting STAT3 and p-STAT3 in the nucleus for IL-6/STAT3 pathway. TNF-α and IL-6 were detected to evaluate the inflammation induced by IH. In mRNA level, there were no statistically significant differences for TNF-α after 4 h, 8 h, and 12 h IH exposure compared with the control group (Fig. 3a), and IL-6 was elevated after 4 h IH exposure, but fell back to initiate level after 8 h and 12 h IH exposure (*P* < 0.05, *F* = 22.058) (Fig. 3b). In protein level for IL-6, there were no statistically significant differences after 4 h, 8 h, and 12 h IH compared with the control group (Fig. 4d). Though we could see the expression of β-catenin in mRNA level was elevated after 12 h IH compared with the control group (*P* < 0.05, *F* = 39.923) (Fig. 3d), no elevation for protein level in the nucleus was observed (Fig. 5c). In another pathway, both mRNA (*P* < 0.05, *F* = 172.351) (Fig. 3c) and protein level (*P* < 0.05, *F* = 40.698) (Fig. 4c) of STAT3 were significantly elevated after 12 h IH compared with the control group. Also, we could observe that p-STAT3 transported to the nucleus was significantly elevated after 2 h IH (*P* < 0.05, *F* = 91.143) (Fig. 5b). These results indicated that STAT3 pathway was activated in IH exposure, which may induce CRC tumorigenesis, but not through IL-6 activating.

### Gut microbiota of OSAS could activate tumorigenesis pathway

**Fig. 6:**
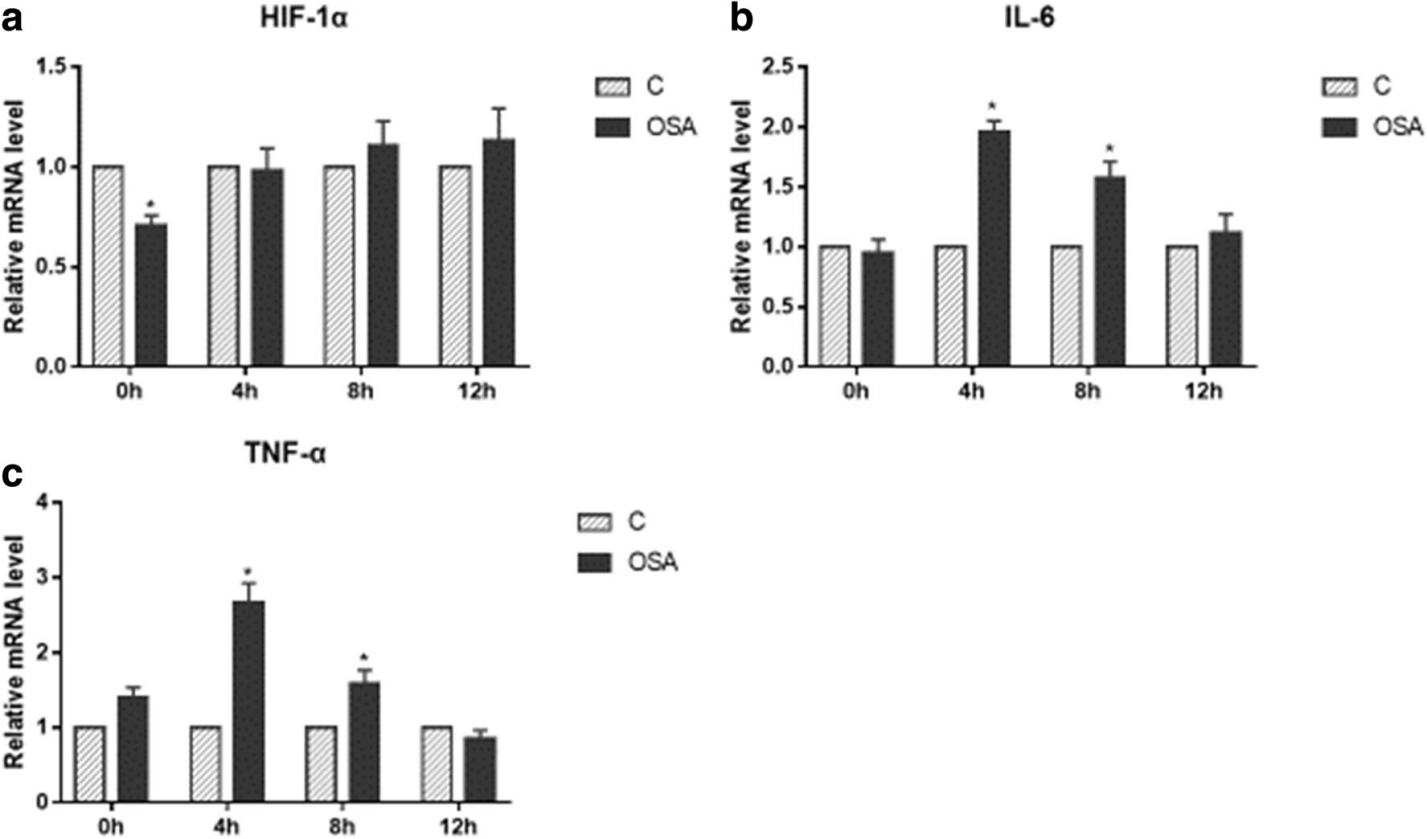
The effect of OSAS fecal content on CRC tumorigenesis pathway under IH condition, mRNA level of HIF-1α (**a**), IL-6 (**b**), and TNF-α (**c**), detected by real-time-PCR. **a** Mann-Whitney test, 0 h group: *P* = 0.037. **b** 4 h group: Mann-Whitney test, *P* = 0.037, 8 h group: *P* = 0.037. **c** 0 h group: Mann-Whitney test, 4 h group: *P* = 0.037, 8 h group: *P* = 0.037. *P < 0.05 vs. C group of each IH time point

**Fig. 7:**
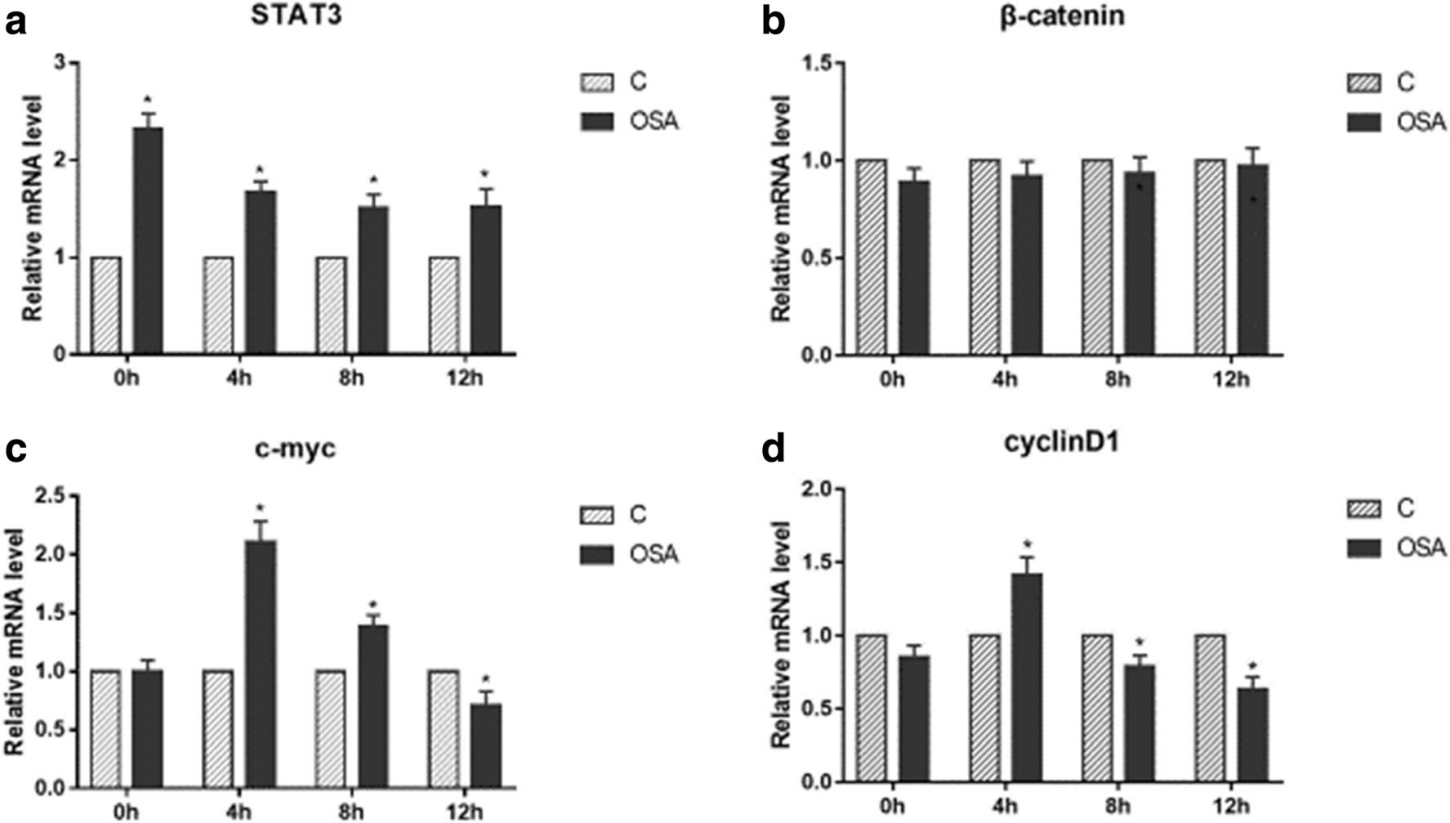
The effect of OSAS fecal content on CRC tumorigenesis pathway under IH condition, mRNA level of STAT3 (**a**), β-catenin (**b**), c-myc (**c**), and cyclinD1 (**d**), detected by real-time-PCR. **a** Mann-Whitney test, 0 h group: *P* = 0.037, 4 h group: *P* = 0.046, 8 h group: *P* = 0.037, 12 h group: *P* = 0.037. **c** Mann-Whitney test, 4 h group: *P* = 0.037, 8 h group: *P* = 0.037, 12 h group: *P* = 0.037. **d** Mann-Whitney test, 4 h group: *P* = 0.046, 8 h group: *P* = 0.037, 12 h group: *P* = 0.037. *P < 0.05 vs. C group of each IH time point

**Fig. 8:**
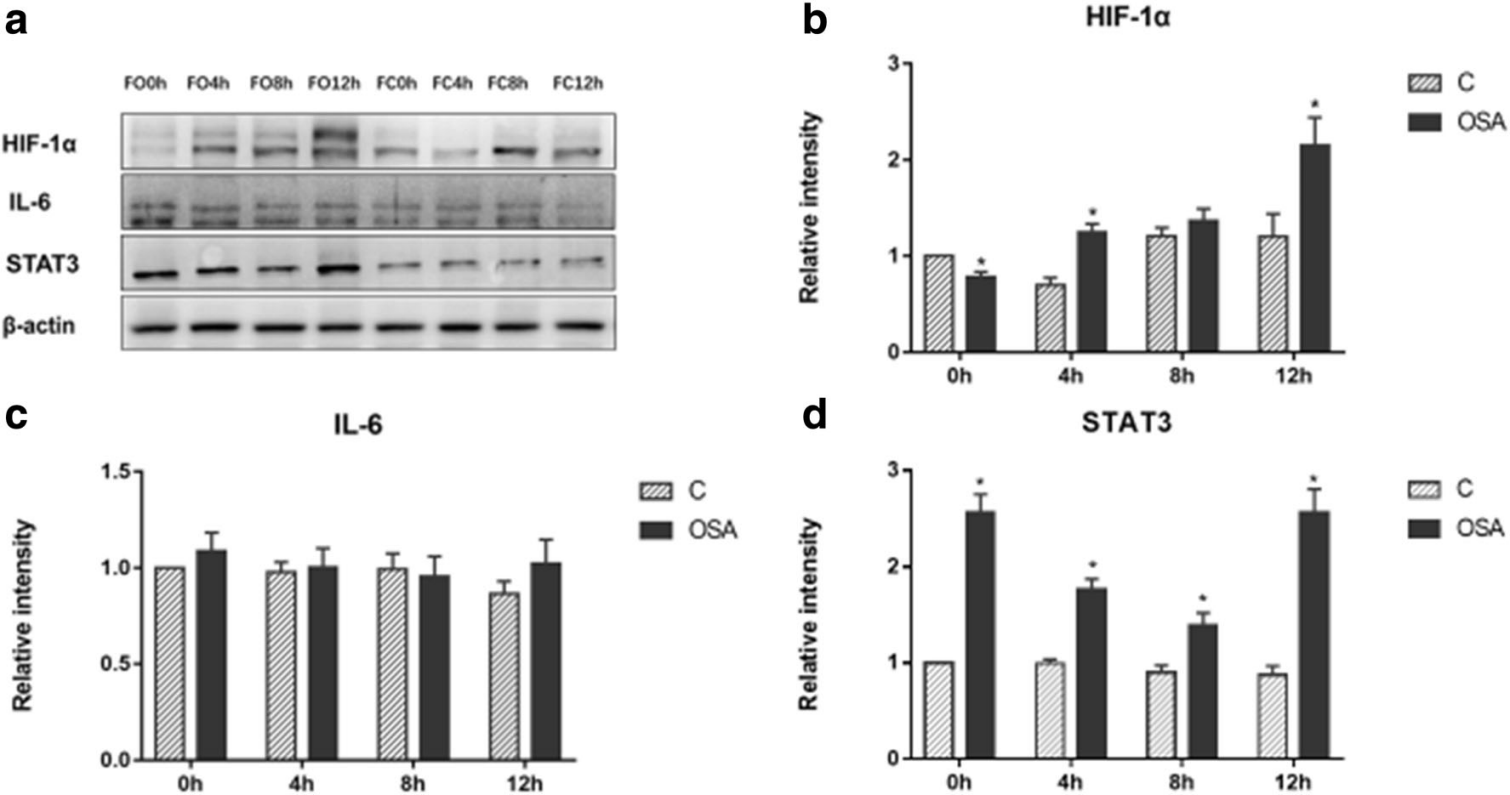
The effect of OSAS fecal content on CRC tumorigenesis pathway under IH condition, detected by Western blot analysis (**a**): the protein level HIF-1α (**b**), IL-6 (**c**), STAT3 (**d**). **b** Mann-Whitney test, 0 h group: *P* = 0.046, 4 h group: *P* = 0.046, 12 h group: *P* = 0.046. **d** Mann-Whitney, 0 h group: *P* = 0.037, 4 h group: *P* = 0.046, 8 h group: *P* = 0.049,12 h group: *P* = 0.037. *P < 0.05 vs. C group of each IH time point

**Fig. 9:**
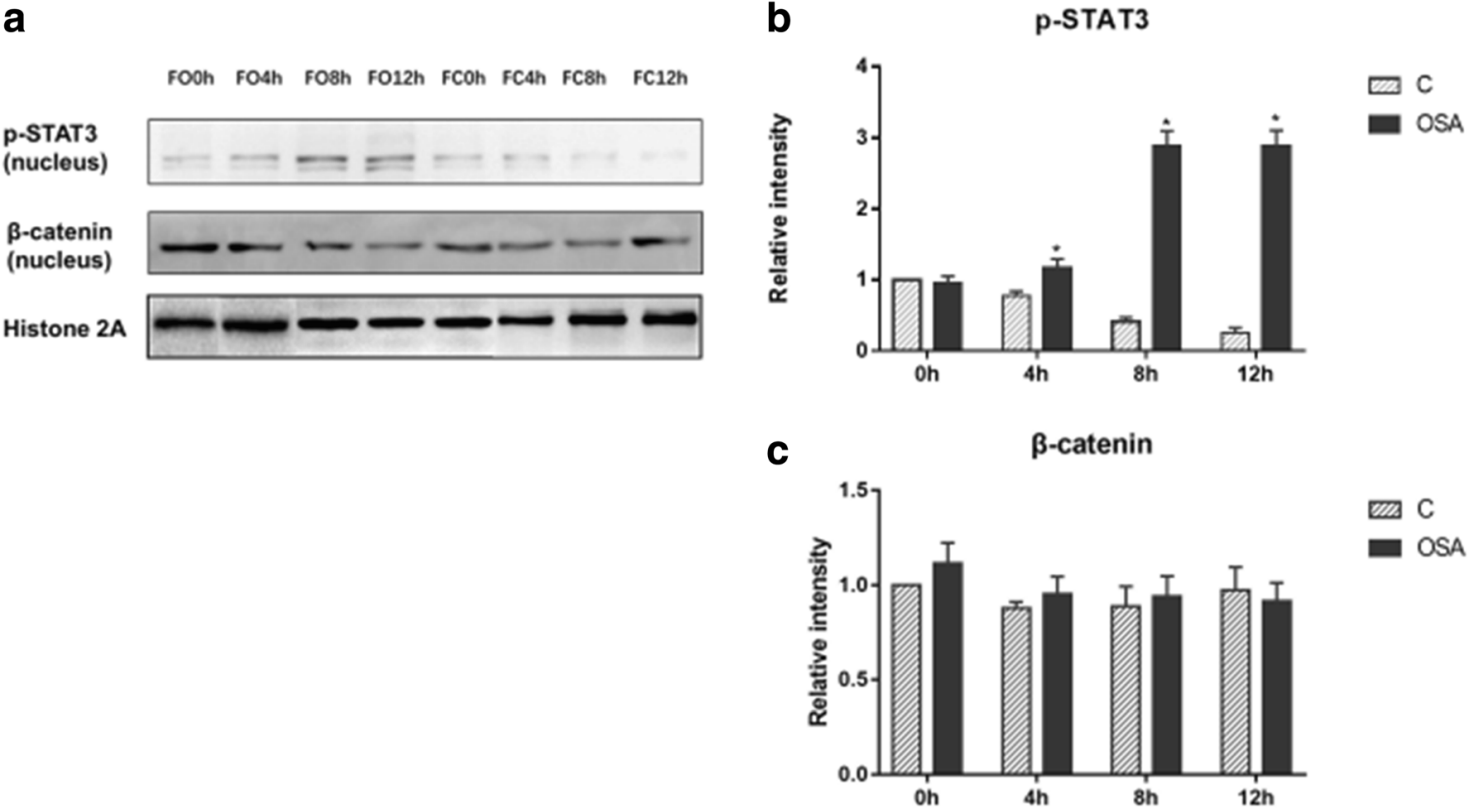
The effect of OSAS fecal content on CRC tumorigenesis pathway under IH condition, detected by Western blot analysis (**a**): the protein level p-STAT3 (**b**), β-catenin (**c**) in IMCE cell nucleus. **b** Mann-Whitney test, 4 h group: *P* = 0.049, 8 h group: *P* = 0.046,12 h group: *P* = 0.046. *P < 0.05 vs. C group of each IH time point

At the mRNA level , we could see O group expressed similar amount mRNA of HIF-1α with C group at most time point, only decreased at 0 h point compared with C group (*P* < 0.05, *F* = 4.528) (Fig. 6a). At the protein level, we could see that the IMCE cells of O group produced lower HIF-1α in the normoxia environment (0 h point, *P* < 0.05, *F* = 7.999) (Fig. 8b), but after IH exposure, O group IMCE cells produced more HIF-1α at 4 h (*P* < 0.05, *F* = 0.131) and 12 h (*P* < 0.05, *F* = 1.252) IH points, and at 8 h IH point, O group’s HIF-1α also had increasing trend but without statistical significance (Fig. 8b). IL-6 was elevated at 4 h (*P* < 0.05, *F* = 13.571) and 8 h (*P* < 0.05, *F* = 6.664) IH points for mRNA level in O group (Fig. 6b), but for protein level, the elevation appeared at 12 h in O group (*P* < 0.05, *F* = 2.937) (Fig. 8c). TNF-α was elevated in O group at 4 h and 8 h points for mRNA level (4 h: *P* < 0.05, *F* = 5.808; 8 h: *P* < 0.05, *F* = 7.942) (Fig. 6c). We could see that both IL-6 and TNF-α were elevated most in mRNA level for 4 h O group, which indicated that the inflammation induced by OSAS patient’s microbiota under IH condition was not proportional to IH duration. The expression of β-catenin in O group had no statistical significance with that in C group at each point both for mRNA level (Fig. 7b) and protein level in the nucleus (Fig. 9c). STAT3 was significantly elevated in O group at each time point for the mRNA level (*P* < 0.05, 0 h: *F* = 5.851; 4 h: *F* = 8.140; 8 h: *F* = 13.795; 12 h: *F* = 12.544) (Fig. 7a) and protein level (*P* < 0.05, 0 h: *F* = 5.325; 4 h: *F* = 7.566; 8 h: *F* = 1.029; 12 h: *F* = 2.444) (Fig. 8d). For p-STAT3 in the nucleus, O group had similar p-STAT3 level with C group at 0 h point, but after IH exposure, more p-STAT3 was detected in O group cell nucleus (*P* < 0.05, 4 h: *F* = 1.569; 8 h: *F* = 13.130; 12 h: *F* = 9.730) (Fig. 9b). These results suggested that gut microbiota of OSAS patients could activate STAT3 pathway under IH condition, which may induce CRC tumorigenesis, and this procedure was not through IL-6 activating. We also could see the c-myc was elevated in O group at 4 h (*P* < 0.05, *F* = 15.246) and 8 h (*P* < 0.05, *F* = 9.446) IH point (Fig. 7c), and cyclinD1 was elevated in O group at 4 h IH point (*P* < 0.05, *F* = 12.714) (Fig. 7d), which indicated that gut microbiota of OSAS patients may activate the cell proliferation under 4 h IH conditions, but not for excessive long or short IH conditions.

## Discussion

MCE cell line is phenotypically normal, but it has proven to be susceptible to transformation, so it is a very suitable model system available for studying malignant progression in colon cancer [56]. In our study, we exposed IMCE cells to IH for 4, 8, and 12 h in the first part. It was observed that the expression of HIF-1α, cyclinD1, and STAT3 was upregulated in the IH group, and p-STAT3 moving into the nucleus was also elevated in the IH group, and both of them getting to the most after 12 h IH; the mRNA of β-catenin was elevated, but the β-catenin protein moving into the nucleus showed no significant change under IH. IL-6 and TNF-α also showed no significant change under IH. Then, we combined the IH exposure and fecal fluid incubation together to treat IMCE cells. Data showed that HIF-1α, STAT3, and p-STAT3 in the nucleus were elevated in the OSAS group compared with the healthy control group. Expressions of IL-6 and TNF-α were elevated in the OSAS group for mRNA level at 4 h and 8 h IH exposure, though without significant change for protein level. The expression of β-catenin in mRNA level and β-catenin protein moving into the nucleus also showed no significant changes between the two groups. The expressions of cyclinD1 and c-myc were upregulated in the OSAS group significantly at 4 h IH exposure, but downregulated in the OSAS group at 12 h IH exposure.

Cancer cells proliferate quickly and consume much oxygen, so malignant tumors usually contain hypoxic regions, which is the characteristic of tumor microenvironment. Tumor cells develop corresponding mechanisms for hypoxia environment adaptation. HIFs are activated in hypoxia conditions, regulating the expression of many genes that code for proteins involved in angiogenesis [57], glucose metabolism, and cell proliferation [58], which play important role for hypoxia tolerance. HIF-1α is the most important one among HIFs. Overexpression of HIF-1α in tumor cells is closely connected with increased resistance to radio- and chemotherapies, increased risk of metastasis, more aggressive phenotype, and strengthened immune suppression [59]. HIFs are also involved in COX2/mPGES-1/PGE2, WNT, and STAT3 signaling pathways correlating with CRC carcinogenesis [25, 60, 61], and implicated in CRC development and metastasis [62–67]. But the conclusions above were acquired under continuous hypoxia condition.

Chronic IH is the key pathogenesis of OSAS damage. Different from continuous hypoxia, IH is in cycling reoxygenation, and reactive oxygen species could be generated in this progress, which is more similar with ischemia-reperfusion process, so OSAS could be regarded as an oxidative stress disorder [68]. The shift frequency of hypoxia and reoxygenation in OSAS patients could be very high, up to more than 5–100 times per hour [69], and blood oxygen saturation may be changed violently, even down to 20%. Multiple organs in OSAS patients would show damage in function and tissue, such as the cardiovascular, brain, liver, pancreas, and kidney. OSAS could aggravate the hypoxia in tissues. But seldom research focused on whether OSAS would promote CRC carcinogenesis. In HCT 116 cells, HIF-1α could be activated in a hypoxia dose-dependent manner under IH condition [70], but whether the pathway correlating with CRC carcinogenesis could be activated under chronic IH condition is still not clear. Our data showed that IH could promote p-STAT3 elevated in the nucleus, and mRNA expression of cyclinD1 also increased at the same time as its downstream target gene, suggesting that STAT3 may be activated under IH condition.

Beside genetic heredity, CRC could be divided into sporadic CRC and colitis-associated cancer (CAC), according to the molecular mechanism by which cancer was triggered and promoted. For sporadic CRC, classical “normal mucosa-adenoma-carcinoma” sequence has been considered to be the core mechanism, and Wnt/β-catenin pathway plays an important role. Once activated, β-catenin would move into the nucleus, regulating its target gene such as cylinD1, c-myc, and MMP-7 which control the cell proliferation [71]. But for CAC, chronic inflammation is a crucial cancer promoter. Two key genes in the inflammatory process, cyclooxygenase-2 (COX-2) and nuclear factor kappaB (NF-kappaB), provide a mechanistic link between inflammation and cancer, while some cytokines, such as TNF-α and IL-6-induced signaling, have been recently shown to promote tumor growth in experimental models of colitis-associated cancer [72]. IL-6 could activate STAT3, which regulates the transcription of regulators of cellular proliferation (cyclinD1, proliferating cell nuclear antigen), survival (BCL-xL, surviving), and angiogenesis (VEGF) [73]. The activation of STAT3 signal pathway has been proved to be key in the CAC carcinogenesis process [73]. How about the mechanism of CRC carcinogenesis correlated with OSAS patients is still unknown to date. Our data showed that STAT3 pathway may be involved in this process. Because STAT3 mediates signaling from multiple inflammation cytokines, not only IL-6, but also IL-11, IL-21, and IL-22, all of which play roles in CRC development [74–80], so the activated STAT3 suggested that chronic inflammation may be involved in this process.

At present, it has been proved that hypoxia and HIFs could influence both Wnt/β-catenin and IL-6/STAT3 pathways. HIF-1 could activate and maintain the Wnt/β-catenin pathway [81]. In HCT 116 cells, Wnt/β-catenin pathway and cell proliferation are inhibited after HIF-1α was blocked [82]; hypoxia could downregulate the APC expression in mRNA and protein level through HIF-1α-dependent mechanism [83]. HIF and p-STAT3 are upregulated in mice and human colon cancer cells, and HIF could promote colon cancer cell proliferation by activating JAK-STAT3 pathway [84]. HIF-1α knockdown leads a significant decrease in the expression levels of STAT3 in human colon cells [85]. But the conclusions above were under continuous hypoxia condition and from cancer cells, how about the effect of chronic IH on colon premalignant cells is still unknown. Our results showed that IH could upregulate the expression of β-catenin mRNA, but we did not observe the increase of β-catenin protein in IMCE cell nucleus, and this indicated that Wnt/β-catenin pathway was not activated indeed in our model. Restricted to experiment conditions, we could not test the effect of longer IH duration on β-catenin. IH could elevate the expression of STAT3 and promote p-STAT3 protein entering to the nucleus, which indicated that STAT3 pathway could be activated by IH. But curiously this STAT3 activation seemed not to be through IL-6 in this process, because we did not see the elevation of IL-6 protein during IH exposure.

Many researches have shown that chronic IH could mimic the OSAS pathogenic process. But only IH itself may not be perfectly suitable for intestine research, because intestinal epithelium is inconstant contact with intestinal microbiota in vivo. It has been proved that the function and disease of intestine are affected by microbiota and their metabolic products. Some research has indicated that intestinal microbiota dysbiosis was induced in OSAS patients [34, 86] and OSAS animal models [87], and the similar situation even occurred in oral [88] and lung [89]. This change may play crucial role in pathogenesis process of OSAS-related hypertension [90, 91]. The changed intestinal microbiota by chronic IH could not be reversed even after normoxic recovery [92]. Those experiments above suggested that intestinal microbiota dysbiosis plays important role in OSAS pathogenic process, so it is not perfectly suitable that using IH only to mimic OSAS for intestine research without microbiota.

The microbiota dysbiosis in OSAS patients may correlate to sleep disorders and metabolic comorbidities. OSAS patients with Prevotella enterotype would exhibit worse sleep disruption [93]. Compared with common people, short-chain fatty acid (SCFA)-producing bacteria was decreased in OSAS patients, accompanied by increased pathogens and elevated levels of IL-6 and homocysteine. Stratification analysis revealed that the Ruminococcus enterotype posed the highest risk for patients with OSAS [34]. Research from real-life patients showed that gut microbiota dysbiosis and decreased SCFA in vivo significantly correlated with CRC [94, 95], so we could hypothesize that gut microbiota dysbiosis and SCFA decrease may be involved in the process of CRC carcinogenesis in OSAS patients. In mouse model, IH exposure could induce higher abundance of Firmicutes and a smaller abundance of Bacteroidetes and Proteobacteria phyla; at the level of dominant microbiota families and genera, Prevotella, Paraprevotella, Desulfovibrio, and Lachnospiraceae increased, whereas Bacteroides, Odoribacter, Turicibacter, Peptococcaceae, and Erysipelotrichaceae decreased in the feces [87]. The co-occurrence of Prevotella and Desulfovibrio suggests a mucin-degrading niche [53], because the sulfate which liberated during Prevotella-mediated mucin degradation could back inhibit this process, but Desulfovibrio could remove the sulfate [96, 97]. The lack of mucin on the epithelial layer of the intestine could potentially lead to a significant alteration in intestinal permeability [87]. The enriched bacteria Desulfovibrio reduces sulfate in order to produce hydrogen sulfide (H2S), which has been reported as a possible contributing risk factor of colorectal cancer [98, 99]. It has been proved that IH can directly impair cellular function and increase epithelium permeability [100], and translocation of nonpathogenic bacteria via the transcellular routes would increase under hypoxia [101]. Failure of intestinal epithelium barrier would lead to chronic inflammation [102]. Plasma IL-6 and TNF-α have been proved to increase in patients with OSA [30, 103–107], and this increase of inflammation cytokines was even not improved by CPAP therapy [103, 108, 109], suggesting the inflammation seems not coming from IH only, which may be partially attributed to gut microbiota dysbiosis and destruction of intestinal epithelium barrier.

Our study showed that both IH and gut microbiota from OSAS patients could promote p-STAT3 entering to the nucleus, but we observed no change for β-catenin pathway in this process. It has been proved that gut microbiota dysbiosis of OSAS patients could induce inflammation, but we only observed elevation of inflammation cytokines for mRNA level in our model, which suggests that our cell model is far from perfect.

Our study had many limitations. We did not test the cell proliferation after IH exposure in this study, just tested the genes related to proliferation. Restricted to experiment conditions, it was not accurate for temperature modulation in IH exposure chamber, so the cells were not in good growth conditions during IH exposure, and this would lead to cell viability decrease or even cell death after longtime IH exposure. The outcome would be affected more when IH duration was longer, so we had to restrict IH duration to 12 h at most. Cell proliferation test, such as MTT test, may be meaningless in this condition. The amount of fecal sample was not sufficient enough, so we did not test the composition of feces microbiota in this study. Though we found STAT3 activated, but the pathway upstream is still not clear. Our IH parameters were performed according to previous teamwork [42, 43], and it was the first time used for intestine cells. We did not try any other IH parameters, or the outcome may be different. Conclusions of cell research still need further validations in animal models and clinical researches. So, our findings are very limited, and there is still very long distance from this conclusion to clarify CRC carcinogenesis associated with OSAS.

## Conclusion

In summary, our data demonstrated that IH could activate the expression of HIF-1α, elevate the level of p-STAT3 in the nucleus, and promote the expression of cylinD1 in IMCE cells. Gut microbiota of OSAS patients could increase the expression of STAT3 and elevate the level of p-STAT3 in the nucleus under IH, and promote the expression of IL-6, and TNF-α in mRNA level under IH less than 8 h. Though elevated in mRNA level, β-catenin protein level in the nucleus was not affected by IH treatment and feces from OSAS patients. This suggested that IH, STAT3 pathway, chronic inflammation, and intestinal microbiota dysbiosis may be involved in CRC carcinogenesis correlated with OSAS. But this conclusion is very limited, still long distance to clarify the CRC development.
